# Perineural Electrical Dry Needling and Neural Mobilization for Chemotherapy-Induced Peripheral Neuropathy: Case Report

**DOI:** 10.3390/jcm14072318

**Published:** 2025-03-28

**Authors:** Austin Granger, James Dunning, Ian Young

**Affiliations:** 1 Macon Rehabilitation and Performance, Macon, GA 31210, USA; 2 American Academy of Manipulative Therapy Fellowship in Orthopaedic Manual Physical Therapy, Montgomery, AL 36104, USA; drjamesdunning@gmail.com (J.D.); tybeewellness@gmail.com (I.Y.); 3 Montgomery Osteopractic Physical Therapy & Acupuncture, Montgomery, AL 36106, USA; 4 Tybee Wellness & Osteopractic, Tybee Island, GA 31328, USA

**Keywords:** chemotherapy-induced peripheral neuropathy, dry needling, neuropathic pain, perineural, neural mobilization, physical therapy

## Abstract

**Background:** Chemotherapy-induced peripheral neuropathy (CIPN) affects 20–85% of individuals exposed to neurotoxic chemotherapeutic agents. Perineural electrical dry needling (PEDN) and neural mobilization (NM) interventions may be beneficial in the management of chronic neurogenic pain; however, there is a paucity of research on the efficacy of both interventions for CIPN. **Methods:** Three patients were referred to an outpatient physical therapy clinic with chronic neuropathic pain associated with CIPN. Each underwent PEDN and NM twice weekly until goals were met or progress stalled. The primary outcome measure was the Numeric Pain Rating Scale (NPRS). Secondary outcomes included the Global Rating of Change (GROC) and the Lower Extremity Functional Scale (LEFS). All outcome measures were assessed at evaluation and discharge. **Results:** At discharge, patients A and B exceeded the minimum clinically important difference (MCID) for the primary and secondary outcome measures, indicating decreased neuropathic pain and improved lower extremity function. Patient C improved in all outcome measures but only experienced clinically meaningful changes in the NPRS and LEFS, not the GROC. **Conclusions:** Following 4–8 sessions of PEDN and NM, three patients with CIPN demonstrated clinically meaningful improvements in chronic lower extremity neuropathic pain and function. PEDN and NM may be beneficial in the management of patients presenting with chronic neuropathic pain secondary to CIPN.

## 1. Introduction

### Background

Chemotherapy-induced peripheral neuropathy (CIPN) is a common, dose-dependent adverse effect of neurotoxic chemotherapeutic agents in the treatment of cancer, affecting approximately 20–85% of patients at standard doses [1,2,3,4,5,6]. The underlying mechanisms of CIPN are complex and vary depending on the specific chemotherapy drugs used. Variation in chemotherapeutic agents alongside differing patient comorbidities and genetic predispositions results in a degree of individualism in patient presentation [1,2,4,5,6]. Approximately 30% of those presenting with CIPN continue to have symptoms beyond 6 months after onset. As cancer survival rates continue to improve, CIPN prevalence is anticipated to rise, increasing patient morbidity and healthcare costs [1,2,3,4]. Preventative treatments for CIPN currently do not exist, necessitating avenues for management after chemotherapy treatment has finished [3,4]. Perineural electrical dry needling (PEDN) and neural mobilization (NM) interventions are beneficial in neurogenic conditions (e.g., idiopathic polyneuropathy, carpal tunnel, cervical radiculopathy, and lumbar radiculopathy), but there are no outcome studies on patients with CIPN [7,8,9,10,11,12].

The neurophysiologic mechanisms of CIPN include but are not limited to neuronal damage, ion channel modulation, mitochondrial dysfunction, microtubule disruption, and the release of chemical mediators facilitating an inflammatory response [2,4,5,13,14,15]. Chemotherapeutic agents penetrate the blood–nerve barrier much easier than the blood–brain barrier, binding to the dorsal root ganglion and peripheral axons. Increased neurotoxins in the peripheral nervous system likely damage the dorsal root ganglion and peripheral nerves, resulting in aberrant sensory findings consistent with CIPN [1,14]. The dorsal root ganglion expresses numerous ion channels, and alterations in ion channel expression peripherally may result in increased sensitivity to stress (e.g., catecholamine release) or cold environments [16,17]. Additionally, mitochondrial and microtubule disruption facilitates neuropathic pain and other adverse sensory stimuli by increasing neuronal hyperexcitability and reducing intraepidermal nerve fibers [2,13]. Lastly, inflammatory cytokines (i.e., tumor necrosis factor-alpha, interleukin-1 beta, interleukin-6, and other chemokines) tend to upregulate and contribute to neuropathic pain [14]. These combined physiologic mechanisms underscore the complex pathophysiology of CIPN, resulting in clinical presentations that differ in severity and variability regarding sensory, motor, and autonomic dysfunction.

Clinical signs are predominantly sensory and develop within a glove-and-stocking distribution; however, CIPN sometimes affects motor and autonomic function. Common symptoms include numbness, tingling, and burning in the hands and feet [1,3,6,14]. The burning sensation is best characterized as neuropathic pain and can persist at night, significantly limiting sleep and impairing daytime productivity [18]. Hyperalgesia at the feet may markedly reduce weightbearing tolerance, with functional implications on standing and walking. At the hands, patients may have marked difficulty in pushing, pulling, lifting, carrying, and fine motor skills (i.e., cooking, drawing, and more) [1,3,6,14]. Impaired somatosensory feedback at the feet often results in deficits related to balance, and a physical examination may demonstrate deficits in kinesthetic or proprioceptive sense at the ankles and wrists [19]. Physical therapy may be an underutilized avenue to help ameliorate symptoms and improve functional mobility in patients with impaired sensorimotor function.

Potential treatment methodologies in the physiotherapy setting include PEDN and NM. Dry needling shares parallels with acupuncture despite differences in underlying principles; however, dry needling does not adhere to the principles of traditional Chinese medicine (TCM) [12,20]. Instead, it concerns anatomical structures and the bio-mechanochemical and neurophysiological effects on muscles, tendons, ligaments, and nerves [12,21]. Contemporary dry needling does not claim to affect qi or utilize TCM diagnoses [12]. However, both interventions use the insertion of monofilament, solid needles with electrical stimulation [12,15,22]. The insertion of needles without injectate may improve collateral circulation via angiogenesis, improve conduction velocity, decrease neuro-inflammatory factors, increase mesenchymal stem cell release for neurovascular unit reconstruction, stimulate endogenous opioid release, and reduce hyperalgesia [12,15,22,23,24,25,26,27,28]. Through the stimulation of mechanochemical changes in the periphery, this study investigates the potential effect of PEDN on CIPN in reducing neural sensitivity.

NM typically encompasses neural gliding or tensioning techniques. A fundamental rationale behind implementing NM is that nerves function better in the presence of blood, space, and mobility [7,16]. A 2017 systematic review and meta-analysis concluded that neural mobility exercises may reduce intraneural edema, improve intraneural fluid dispersion, reduce hyperalgesia, and mitigate increased immune response after damage to neural tissue [7]. NM may be useful in the management of various types of neuropathies (e.g., entrapment, diabetic, idiopathic) in improving patient symptoms and functional capacity; however, NM has not been extensively studied in CIPN [7,8].

No prior studies have examined the effects of PEDN and NM in patients with chronic CIPN [28,29]. One case report reported favorable outcomes from the use of PEDN and NM in a single patient with idiopathic peripheral neuropathy [8]. NM may be beneficial for neuropathic pain conditions, but no research has reported outcomes in patients with CIPN [7,8]. Importantly, no clinical practice guidelines exist for CIPN in the physical therapy setting [30]. We speculated that the combination of PEDN and NM may improve pain and disability in patients with CIPN.

## 2. Case Description

### 2.1. Patients

Three individuals presented to Macon Rehabilitation and Performance in Macon, GA., via physician referral between March and December of 2024 and underwent physical therapy management of symptoms related to CIPN. Informed consent was obtained from each patient to perform PEDN and NM treatment interventions. Baseline variables (Table 1) were recorded on the first visit. The time since the preceding chemotherapy treatment was eight, twelve, and eleven months. Patient cancer types consisted of ovarian (*n* = 1) and breast (*n* = 2). The ages of the three patients were 52, 70, and 72. All patients were female. All patients reported no change in their neuropathy symptoms in the three months before beginning physical therapy. Two patients (B and C) had a concurrent history of diabetes mellitus type II. None of the patients had a history of uncontrolled diabetes, foot ulceration, seizures, lumbar radiculopathy, diabetic polyneuropathy, needle aversion, pacemaker use, or bleeding disorders. All three patients presented with neuropathic pain in a glove-and-stocking distribution (i.e., symptoms distal to the wrist and ankle). Patients were seen twice a week until long-term goals were met (*n* = 2) or progress stalled (*n* = 1). Long-term goals for all patients primarily included standing and walking for longer durations to ameliorate household- and community-level mobility deficits.

### 2.2. Treating Clinician

Patients were treated by a physical therapist who had two years of experience and completed a 54 h dry needling certification training course with content specific to perineural needling strategies [31].

### 2.3. Evaluation Procedure

The treating clinician performed a physical examination of each patient, including observation, sensation testing, range of motion testing, and strength testing. The clinical presentation was verified to be consistent with that of the referral. Clinical observation specifically ensured that the patients had no notable areas of redness, swelling, or calluses of the feet. Protective sensation (e.g., Semmes–Weinstein monofilament test) was tested for all patients and was intact. Kinesthesia and proprioception were tested at the level of the ankle and great toe. Active range of motion and strength testing ruled out motor impairment in all three patients.

### 2.4. Outcome Measures

Patients completed the Numeric Pain Rating Scale (NPRS), Lower Extremity Functional Scale (LEFS), and Global Rating of Change (GROC) at evaluation and discharge. The NRPS served as the primary outcome measured, with validity likely improved via the concurrent utilization of the GROC to lessen the effects of recall bias [32]. The NPRS measured average pain intensity over the preceding 24 h at bilateral distal lower extremities specific to CIPN using an 11-point scale from 0 (no pain) to 10 (worst pain imaginable) [33]. It was administered graphically for all subjects. Research supports the NRPS in both chronic nociceptive and neuropathic conditions as a valid and reliable outcome measure for assessing pain intensity over the preceding day, and it is utilized in other studies examining physical therapy for neuropathic pain [8,33,34,35,36,37]. The minimal clinically important difference (MCID) of the NRPS for chronic pain, including neuropathic pain, is a 1.7–2.0-point change [36,37]. Other research utilizing the NRPS for CIPN has reported an MCID of 2.0 secondary to the discrete nature of the scale for clinical relevance [8,35].

Secondary outcome measures included the GROC and LEFS. The GROC scale was presented graphically at each reassessment and had a 15-point range from −7 (a very great deal worse) to +7 (a very great deal better). The existing literature reports meaningful improvement as plus or minus 5 on a 15-point GROC scale for chronic pain conditions; nevertheless, the GROC instrument has been infrequently used for patients with neuropathy [32,38]. Notably, the GROC gives little to no information regarding functional improvements and is best used contextually with other outcome measures [39]. This case study of three patients utilized the GROC alongside the NPRS and LEFS. The LEFS assesses a patient’s lower extremity function and is deemed valid and reliable for numerous patient presentations with an MCID of 9 points [40,41].

### 2.5. Intervention

**Figure 1:**
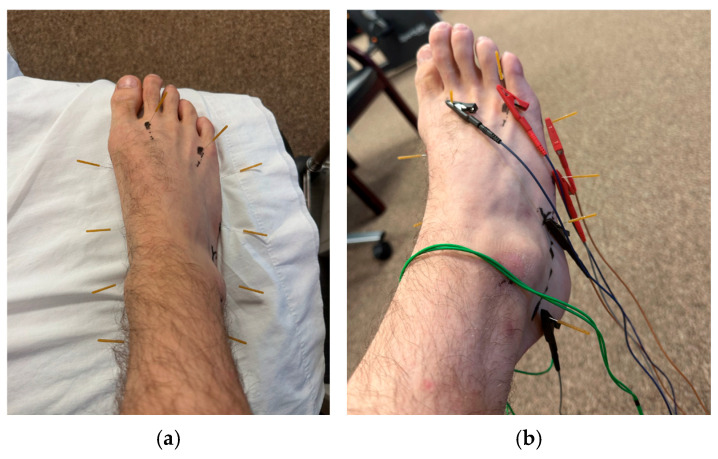
(**a**) Dry needling protocol used for CIPN. (**b**) Dry needling protocol for CIPN with electrical stimulation.

PEDN and NM were chosen as the primary interventions due to the growing research supporting the use of each for neuropathic pain conditions [7,12,15,16,22,23,24,25,26,27,28]. Three patients underwent PEDN and NM twice a week for four, eight, and eight visits, respectively. PEDN was performed with ten 30 mm length by 0.30 mm diameter J-SERIN needles in each lower extremity, separately. Needle locations were consistent among all patients (Figure 1, Table 2).

This protocol emphasized 10 points on each lower extremity, with the target tissues being the tibial, medial, and lateral plantar nerves, sural nerve, intermediate dorsal cutaneous nerve, medial branch of the deep peroneal nerve, and lateral dorsal cutaneous nerve. The needles were left in situ for 20 min on each lower extremity, with manual manipulation via four full 360-degree unidirectional needle rotations every 5 min as tolerated. The ITO^®^ ES-160 was used for electrical stimulation, with a preset program varying frequency from 30 to 100 Hz. Research supports both high and low frequencies but notes that low frequencies may be less tolerable to patients with neuropathic pain [25,42,43]. Pulse duration was set at 150 microseconds for appropriate mechanochemical effects and improved patient tolerance (i.e., less pain) [44]. Intensity was increased until described by the patient as “moderate” [43]. The needling locations did not require adjustment for any of the patients throughout their visits; however, the patients’ perceived intensity of “moderate” seemed to increase in later visits, resulting in a graded increase in the intensity of electrical manipulation.

**Figure 2:**
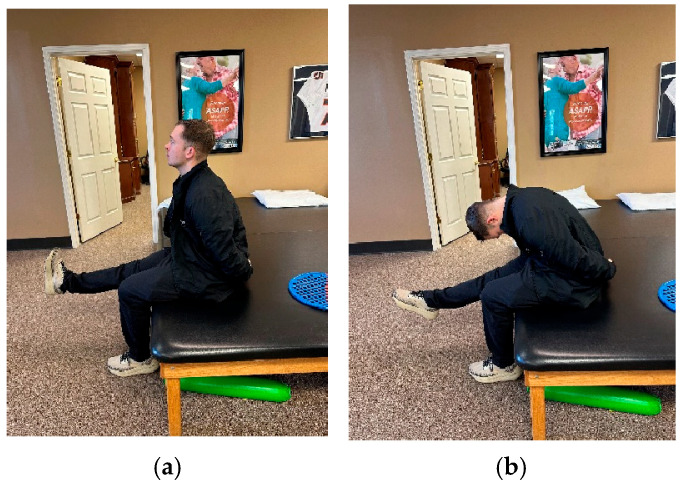
(**a**) Slump sliders (cervical extension coupled with ankle dorsiflexion). (**b**) Slump sliders (cervical flexion coupled with ankle plantarflexion).

NM included slump nerve sliders as tolerated into mild pain or tingling but not numbness [7,11,45]. Patients were positioned in a seated forward lean with cervical flexion and unilateral knee extension. They were then asked to extend their cervical spine in combination with dorsiflexion and flex their cervical spine in combination with plantarflexion, resulting in an excursion of the sciatic nerve and its branches (Figure 2) [46,47]. Sliders facilitate neural mobility by repetitive up- and downglides performed as the sole component of a home exercise program (HEP). In this study, patients were asked to perform NM every hour throughout the day for 30 repetitions on each lower extremity [7,11,45,46,47,48]. Subjective compliance with the HEP was 100% for patients A and B and 50% for patient C.

### 2.6. Treatment Adverse Events

Adverse events were defined as any distressing or intolerable events (e.g., increased redness, pain, or other neuropathic symptoms) lasting longer than a day [49]. No patient had prolonged redness or significant post-needling soreness. No bruising was present at follow-ups for all patients. Therefore, none of the patients had adverse events or required alterations to the treatment plan.

### 2.7. Statistical Analysis

Outcomes were evaluated based on clinically meaningful changes [32,36,37,38,39,40,41]. Due to the limited sample size, inferential statistical tests were not conducted. Changes in outcomes over time compared to MCIDs were used to investigate the clinical utility of PEDN and NM for CIPN.

## 3. Results

All patients were seen by the same physical therapist and took the same treatment approach, utilizing PEDN and NM. All patients lacked a history of peripheral neuropathy prior to the onset of CIPN. Secondary to marked pain reduction and meeting goals of care, patient A completed four visits, while patients B and C completed eight. Patients A and B reported meeting the goals outlined in the plan of care, and patient C had a stall in progress.

**Figure 3:**
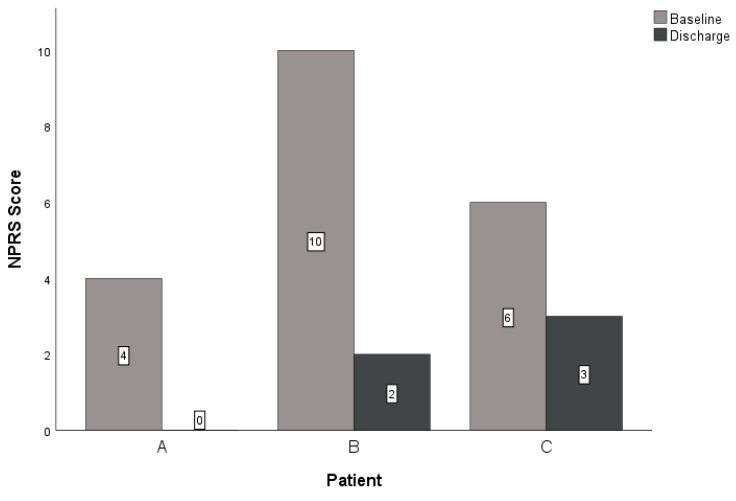
Changes in NPRS. Abbreviations: NPRS—Numeric Pain Rating Scale.

**Figure 4:**
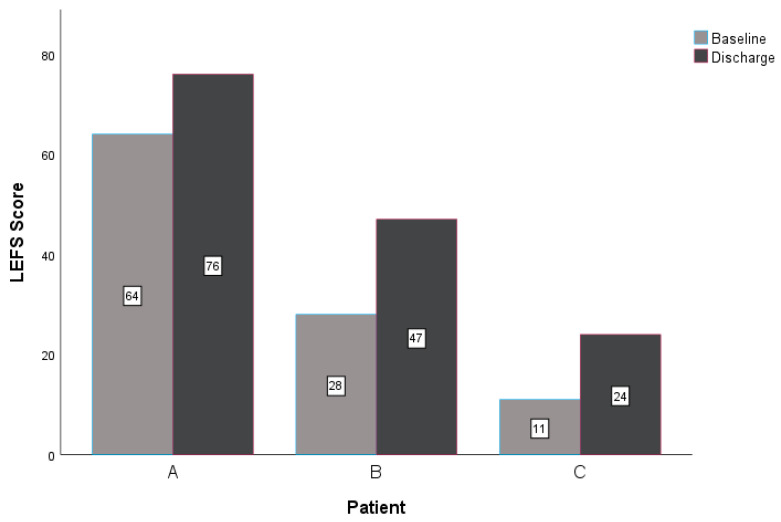
Changes in LEFS. Abbreviations: LEFS—Lower Extremity Functional Scale.

At discharge, all three patients reported improved scores for the NPRS, GROC, and LEFS. The within-group change scores for the NPRS exceeded the MCID for this measure in all three patients (Table 3, Figure 3) [36,37]. All subjects reported notable improvement in the perceived benefit of the intervention for their lower extremity CIPN symptoms. Patient A and patient B exceeded the MCID of greater than or equal to 5 on a 15-point scale (Table 3) [32,38]. Lastly, the LEFS indicated improved tolerance for functional mobility for all patients at discharge. All patients reported a change in the LEFS that exceeded the nine-point MCID (Table 3, Figure 4) [40,41]. All patients demonstrated clinically relevant improvements with care regarding reduced neuropathic pain, subjective reports of improvement in symptoms, and improved function at four and eight visits (if applicable).

## 4. Discussion

Three patients underwent similar treatment plans for bilateral lower extremity CIPN symptoms using PEDN and NM. Upon reassessment, all three patients improved with treatment over time. Patients A and B reported clinically relevant improvements in all outcome measures, and patient C noted clinically relevant improvements in the NPRS and LEFS (Table 3, Figure 3 and Figure 4). These findings are consistent with a similar case report investigating PEDN and NM in patients with idiopathic peripheral neuropathy, supporting the role of perineural needling and neural mobility intervention strategies in neuropathic pain syndromes [8].

PEDN and NM likely affect neuropathic symptomology through numerous neurophysiologic mechanisms. PEDN has been found to stimulate the increased release of corticosterone, calcitonin gene-related peptide (CGRP) at low quantities, mesenchymal stem cells, norepinephrine, and beta-endorphins, alongside facilitating angiogenesis and subsequent neuronal regeneration and repair [8,14,15,21,22,23,27]. This is consistent with other studies noting marked increases in extra- and intraneural circulation and neovascularization [12,21,22,24]. Additionally, the upregulation of inflammatory cytokines plays a notable role in CIPN symptom severity, and PEDN may have an inhibitory effect on this process [14,21,23]. PEDN, with a combination of mechanical and electrical manipulation in situ, stimulates a 10-fold increase in corticosterone and marked increases in norepinephrine and beta-endorphin release through the stimulation of the sympathetic nervous system [21]. Each of these chemicals functionally inhibits the production of inflammatory cytokines. Additionally, PEDN increases CGRP, which may have an anti-inflammatory effect through tetrodotoxin inhibition at low quantities [21]. Lastly, PEDN may reduce cortical sensitization associated with chronic neuropathic pain through the stimulation of endogenous opioid release and modulation of calcium ion activity within the primary somatosensory cortex [24,50]. As nerves function best in the presence of blood, space, and mobility, the above neuroendocrine changes secondary to PEDN have the potential to benefit from increased angiogenesis noted in electro-needling interventions. These processes facilitate an improved chemical environment conducive to neuronal regeneration and a reduction in hyperalgesia [8,12,16,21,22,24,26,51].

Similarly, NM may modulate neuropathic pain and improve neuronal sensory function through intraneural and extraneural mechanisms. NM may facilitate nerve regeneration by reducing intraneural edema, increasing intraneural fluid dispersion, reducing hyperalgesia, and ameliorating increased inflammatory factors [7,48,52,53]. In addition, NM may improve neural mobility in the lower extremities; however, there is a lack of data investigating neural excursion in CIPN [46,47]. However, prior studies investigating peripheral neurogenic pain have found decreased neural excursion in the affected sample, and symptom improvement was correlated with improved neural mobility and reduced H-reflex latency (i.e., indicating improved nerve conductivity) [9,10,54]. Further research would be beneficial in elucidating the therapeutic mechanisms of NM in CIPN.

Although no cause-and-effect relationships can be identified from case reports, this is the first study to report the clinical utility of PEDN and NM in patients with CIPN, to the authors’ knowledge. Existing research for other neuropathic conditions is also limited, but studies with a lower level of evidence do exist, examining one or both of these interventions in conditions including but not limited to idiopathic peripheral neuropathy, infrapatellar saphenous neuralgia, cervical radiculopathy, and carpal tunnel [7,8,9,10,11,12,54,55]. Therefore, future research should involve a two-arm randomized clinical trial to investigate any cause-and-effect relationships pertaining to PEDN and NM with symptoms associated with CIPN.

### Limitations

This report is of a lower level of evidence, as it cannot establish causal relationships and lacks contextual information to explain discrepancies in patient presentation [56]. Additionally, there is insufficient contextual knowledge regarding specific chemotherapeutic medications and duration, with recall deficits being a potential contributing factor. This information could have provided valuable insight into the initial patient presentation and patients’ responsiveness to treatment. The results may also be limited in generalizability to other chronic neuropathic conditions. Nevertheless, physical therapy treatment is largely multimodal, and this study examined the effect of a combination of two interventions on patient outcomes. Therefore, this article can only report that the combination of PEDN and ND facilitated clinically meaningful changes and cannot quantify the extent to which each impacted progress.

## 5. Conclusions

This case series highlights the potential therapeutic benefit of adding PEDN and NM to treatment protocols for patients suffering with chronic neuropathic pain secondary to CIPN. Randomized controlled trials will be beneficial in establishing causal inference and generating effect sizes.

## Figures and Tables

**Table 1:** Baseline variables: demographics and outcome measures.

Baseline Variable	Patient A	Patient B	Patient C
Age (Years)	52	70	72
Sex	Female	Female	Female
Time Since Onset (Months)	8	12	11
Diabetic	No	Yes	Yes
History of Diabetic Peripheral Neuropathy	No	No	No
BMI	30.8	20.9	31.5
NPRS	4	10	6
LEFS	64	28	11

Abbreviations: BMI—Body Mass Index; NPRS—Numeric Pain Rating Scale, 0–10, lower scores equate to decreased subjective pain severity over the preceding 24 h; LEFS—Lower Extremity Functional Scale, 0–80, lower scores indicate decreased patient function.

**Table 2:** Needle points.

Anatomy/Target Tissue	Location	Needle Angulation
Lateral dorsal cutaneous nerve	Within the depression midway between the prominence of the lateral malleolus and Achilles tendon	Medial
Lateral dorsal cutaneous nerve	In the depression just distal to the apex of the lateral malleolus	A-P or P-A
Lateral dorsal cutaneous nerve	Within the depression just plantar and distal to the base of the 5th metatarsal	Medial
Lateral dorsal cutaneous nerve	Within the depression just plantar and proximal to the head of the 5th metatarsal	Medial
Tibial nerve	Halfway between the medial malleolus and Achilles tendon	Perpendicular
Proximal belly of abductor hallucis and medial plantar nerve	In the depression just medial to the navicular tuberosity	Medial to Lateral
Medial aspect of distal plantar aponeurosis, medial plantar nerve, and neurovascular supply to medial foot	In the depression distal and inferomedial to the base of the 1st metatarsal	Medial to Lateral
Medial aspect of distal plantar aponeurosis, medial plantar nerve, and neurovascular supply to medial foot	In the depression proximal and inferomedial to the 1st metatarsophalangeal joint	Medial to Lateral
1st dorsal interossei, medial branch of deep peroneal nerve, and dorsalis pedis artery	Dorsum of foot, within 1st dorsal interosseus, in the depression just distal to the junction of the 1st and 2nd metatarsal bones	Oblique Distal
Intermediate dorsal cutaneous nerve and dorsolateral aspect of distal plantar aponeurosis	In the web space between the 4th and 5th toes	Perpendicular

**Table 3:** Outcome assessments.

	Baseline Score	Discharge Score	Net Change Score
Patient A			
NPRS	4	0	−4
LEFS	64	76	+12
GROC	-	+6	+6
Patient B			
NPRS	10	2	−8
LEFS	28	47	+19
GROC	-	+5	+5
Patient C			
NPRS	6	3	−3
LEFS	11	24	+13
GROC	-	+3	+3

Abbreviations: NPRS—Numeric Pain Rating Scale, 0–10, lower scores equate to decreased subjective pain severity over the last 24 h; LEFS—Lower Extremity Functional Scale, 0–80, lower scores indicate decreased patient function; GROC—Global Rating of Change, −7 to +7.

## Data Availability

The original contributions presented in the study are included in the article, further inquiries can be directed to the corresponding authors.

## References

[1] Bae E.H., Greenwald M.K., Schwartz A.G. (2021). Chemotherapy-induced peripheral neuropathy: Mechanisms and therapeutic avenues. Neurotherapeutics.

[2] Chua K.C., El-Haj N., Priotti J., Kroetz D.L. (2022). Mechanistic insights into the pathogenesis of microtubule-targeting agent-induced peripheral neuropathy from pharmacogenetic and functional studies. Basic Clin. Pharmacol Toxicol..

[3] Colvin L.A. (2019). Chemotherapy-induced peripheral neuropathy: Where are we now?. Pain.

[4] Staff N.P., Grisold A., Grisold W., Windebank A.J. (2017). Chemotherapy-induced peripheral neuropathy: A current review. Ann Neurol..

[5] Was H., Borkowska A., Bagues A., Tu L., Liu J.Y.H., Lu Z., Rudd J.A., Nurgali K., Abalo R. (2022). Mechanisms of chemotherapy-induced neurotoxicity. Front. Pharmacol..

[6] Zajączkowska R., Kocot-Kępska M., Leppert W., Wrzosek A., Mika J., Wordliczek J. (2019). Mechanisms of chemotherapy-induced peripheral neuropathy. Int. J. Mol. Sci..

[7] Basson A., Olivier B., Ellis R., Coppieters M., Stewart A., Mudzi W. (2017). The effectiveness of neural mobilization for neuromusculoskeletal conditions: A systematic review and meta-analysis. J. Orthop. Sports Phys. Ther..

[8] Nasr A.J., Zafereo J. (2019). The effects of dry needling and neurodynamic exercise on idiopathic peripheral neuropathy: A case report. J. Bodyw. Mov. Ther..

[9] Thoomes E., Ellis R., Dilley A., Falla D., Thoomes-de Graaf M. (2021). Excursion of the median nerve during a contra-lateral cervical lateral glide movement in people with and without cervical radiculopathy. Musculoskelet Sci. Pract..

[10] Hegazy M.M., Gomaa E.F., Abd El Mageed S.F., El Habashy H.R. (2019). H-reflex latency changes after combined application of traction and neural mobilization in cervical radiculopathy. Egypt. J. Neurol. Psychiatr. Neurosurg..

[11] Baptista F.M., Nery E., Cruz E.B., Afreixo V., Silva A.G. (2024). Effectiveness of neural mobilisation on pain intensity, functional status, and physical performance in adults with musculoskeletal pain—A systematic review with meta-analysis. Clin. Rehabil..

[12] Dunning J., Butts R., Mourad F., Young I., Flannagan S., Perreault T. (2014). Dry needling: A literature review with implications for clinical practice guidelines. Phys Ther Rev..

[13] Doyle T.M., Salvemini D. (2021). Mini-Review: Mitochondrial dysfunction and chemotherapy-induced neuropathic pain. Neurosci. Lett..

[14] Wang X.M., Lehky T.J., Brell J.M., Dorsey S.G. (2012). Discovering cytokines as targets for chemotherapy-induced painful peripheral neuropathy. Cytokine.

[15] Salazar T.E., Richardson M.R., Beli E., George J., Kim Y., Duan Y., Moldovan L., Yan Y., Bhatwadekar A., Jadhav V. (2017). Electroacupuncture promotes central nervous system-dependent release of mesenchymal stem cells. Stem Cells.

[16] Louw A., Puentedura E., Schmidt S., Zimney K. (2018). Pain Neuroscience Education: Teaching People About Pain.

[17] Cuenca-Zaldívar J.N., del Corral-Villar C., García-Torres S., Araujo-Zamora R., Gragera-Peña P., Martínez-Lozano P., Sánchez-Romero E.A. (2025). Fourteen-year retrospective cohort study on the impact of climatic factors on chronic musculoskeletal pain: A Spanish primary care analysis. Int. J. Rheum Dis..

[18] Mahfouz F.M., Li T., Joda M., Harrison M., Horvath L.G., Grimison P., King T., Marx G., Goldstein D., Park S.B. (2024). Sleep dysfunction associated with worse chemotherapy-induced peripheral neurotoxicity functional outcomes. Support. Care Cancer.

[19] Shah V.V., Muzyka D., Guidarelli C., Sowalsky K., Horak F.B., Winters-Stone K.M. (2024). Chemotherapy-induced peripheral neuropathy and falls in cancer survivors relate to digital balance and gait impairments. JCO Precis. Oncol..

[20] Dommerholt J. (2011). Dry needling—Peripheral and central considerations. J. Man Manip. Ther..

[21] Butts R., Dunning J. (2016). Peripheral and spinal mechanisms of pain and dry needling mediated analgesia: A clinical resource guide for health care professionals. Int. J. Phys. Med. Rehabil..

[22] Jin Z.-F., Shi L., Cao H.-M., Li Y., Xu S.-X., Zhang Y. (2017). Electroacupuncture improves neurovascular unit reconstruction by promoting collateral circulation and angiogenesis. Neural Regen Res..

[23] Cha M.H., Nam T.S., Kwak Y., Lee H., Lee B.H. (2012). Changes in cytokine expression after electroacupuncture in neuropathic rats. Evidence-Based Complement. Altern. Med..

[24] Zhou M., Zhang Q., Huo M., Song H., Chang H., Cao J., Fang Y., Zhang D. (2023). The mechanistic basis for the effects of electroacupuncture on neuropathic pain within the central nervous system. Biomed Pharmacother..

[25] Zheng Y., Jiang M., Wei Z., Chi H., Kang Y., Li S., Zheng Y., He X., Shao X., Fang J. (2024). Electroacupuncture alleviates neuropathic pain in a rat model of CCD via suppressing P2 × 3 expression in dorsal root ganglia. Chin. Med..

[26] Hsiao I.H., Yen C.M., Hsu H.C., Liao H.Y., Lin Y.W. (2024). Chemogenetics modulation of electroacupuncture analgesia in mice spared nerve injury-induced neuropathic pain through TRPV1 signaling pathway. Int. J. Mol. Sci..

[27] Liu Y.-P., Luo Z.-R., Wang C., Cai H., Zhao T.-T., Li H., Shao S.-J., Guo H.-D. (2020). Electroacupuncture promoted nerve repair after peripheral nerve injury by regulating miR-1b and its target brain-derived neurotrophic factor. Front. Neurosci..

[28] Chien T.J., Liu C.Y., Fang C.J., Kuo C.Y. (2019). The efficacy of acupuncture in chemotherapy-induced peripheral neuropathy: Systematic review and meta-analysis. Integr Cancer Ther..

[29] Bao T., Patil S., Chen C., Zhi I.W., Li Q.S., Piulson L., Mao J.J. (2020). Effect of acupuncture vs. sham procedure on chemotherapy-induced peripheral neuropathy symptoms. JAMA Netw Open..

[30] Stoller S., Capozza S., Alberti P., Lustberg M., Kleckner I.R. (2023). Framework to leverage physical therapists for the assessment and treatment of chemotherapy-induced peripheral neurotoxicity (CIPN). Support. Care Cancer.

[31] American Academy of Manipulative Therapy DN-2: Dry Needling for Lumbopelvic & Lower Extremity Conditions: An Evidence-Based Approach. https://spinalmanipulation.org/dates-and-locations/dn-2-dry-needling/.

[32] Bobos P., Ziebart C., Furtado R., Lu Z., MacDermid J.C. (2020). Psychometric properties of the global rating of change scales in patients with low back pain, upper and lower extremity disorders. A systematic review with meta-analysis. J. Orthop..

[33] Jensen M.P., McFarland C.A. (1993). Increasing the reliability and validity of pain intensity measurement in chronic pain patients. Pain.

[34] Alghadir A., Anwer S., Iqbal A., Iqbal Z. (2018). Test-retest reliability, validity, and minimum detectable change of visual analog, numerical rating, and verbal rating scales for measurement of osteoarthritic knee pain. J. Pain Res..

[35] Andersen Hammond E., Pitz M., Steinfeld K., Lambert P., Shay B. (2020). An exploratory randomized trial of physical therapy for the treatment of chemotherapy-induced peripheral neuropathy. Neurorehabil Neural Repair..

[36] Farrar J.T., Young J.P., LaMoreaux L., Werth J.L., Poole M.R. (2001). Clinical importance of changes in chronic pain intensity measured on an 11-point numerical pain rating scale. Pain.

[37] Hawker G.A., Mian S., Kendzerska T., French M. (2011). Measures of adult pain: Visual Analog Scale for Pain (VAS Pain), Numeric Rating Scale for Pain (NRS Pain), McGill Pain Questionnaire (MPQ), Short-Form McGill Pain Questionnaire (SF-MPQ), Chronic Pain Grade Scale (CPGS), Short Form-36 Bodily Pain Scale (SF-36 BPS), and Measure of Intermittent and Constant Osteoarthritis Pain (ICOAP). Arthr. Care Res..

[38] Kamper S.J., Maher C.G., Mackay G. (2009). Global Rating of Change scales: A review of strengths and weaknesses and considerations for design. J. Man. Manip. Ther..

[39] Schmitt J., Abbott J.H. (2015). Global ratings of change do not accurately reflect functional change over time in clinical practice. J. Orthop. Sports Phys. Ther..

[40] Mehta S.P., Fulton A., Quach C., Thistle M., Toledo C., Evans N.A. (2016). Measurement properties of the Lower Extremity Functional Scale: A systematic review. J. Orthop. Sports Phys. Ther..

[41] Zhang Y., Zang Y., Martin R.L. (2024). Clinically most relevant psychometric properties of the Lower Extremity Functional Scale: A systematic review. Disabil. Rehabil..

[42] Huang J., Yang C., Zhao K., Zhao Z., Chen Y., Wang T., Qu Y. (2022). Transcutaneous electrical nerve stimulation in rodent models of neuropathic pain: A meta-analysis. Front. Neurosci..

[43] Sluka K.A., Walsh D. (2003). Transcutaneous electrical nerve stimulation: Basic science mechanisms and clinical effectiveness. J. Pain.

[44] Dalamagka M.I. (2023). Acupuncture and electrotherapy: An alternative and complementary treatment method. World J. Adv. Res. Rev..

[45] Ellis R.F., Hing W.A. (2008). Neural mobilization: A systematic review of randomized controlled trials with an analysis of therapeutic efficacy. J. Man. Manip. Ther..

[46] Alshami A.M., Alshammari T.K., AlMuhaish M.I., Hegazi T.M., Tamal M., Abdulla F.A. (2022). Sciatic nerve excursion during neural mobilization with ankle movement using dynamic ultrasound imaging: A cross-sectional study. J. Ultrasound..

[47] Ellis R.F., Hing W.A., McNair P.J. (2012). Comparison of longitudinal sciatic nerve movement with different mobilization exercises: An in vivo study utilizing ultrasound imaging. J. Orthop. Sports Phys. Ther..

[48] Gilbert K.K., James C.R., Apte G., Brown C., Sizer P.S., Brismée J.-M., Smith M.P. (2015). Effects of simulated neural mobilization on fluid movement in cadaveric peripheral nerve sections: Implications for the treatment of neuropathic pain and dysfunction. J. Man. Manip. Ther..

[49] Carlesso L.C., MacDermid J.C., Santaguida L.P. (2010). Standardization of adverse event terminology and reporting in orthopaedic physical therapy: Application to the cervical spine. J. Orthop. Sports Phys. Ther..

[50] Wei J.-A., Hu X., Zhang B., Liu L., Chen K., So K.-F., Li M., Zhang L. (2021). Electroacupuncture activates inhibitory neural circuits in the somatosensory cortex to relieve neuropathic pain. iScience.

[51] Zimney K., Van Bogaert W., Louw A. (2023). The biology of chronic pain and its implications for pain neuroscience education: State of the art. J. Clin. Med..

[52] Carta G., Fornasari B.E., Fregnan F., Ronchi G., De Zanet S., Muratori L., Nato G., Fogli M., Gambarotta G., Geuna S. (2022). Neurodynamic treatment promotes mechanical pain modulation in sensory neurons and nerve regeneration in rats. Biomedicines.

[53] Schmid A.B., Brunner F., Luomajoki H., Held U., Bachmann L.M., Künzer S., Coppieters M.W. (2009). Reliability of clinical tests to evaluate nerve function and mechanosensitivity of the upper limb peripheral nervous system. BMC Musculoskelet. Disord..

[54] Burgess N.E., Gilbert K.K., Sobczak S., Sizer P.S., Homen D., Lierly M., Kearns G.A., Brismée J.-M. (2023). Upper limb neurodynamic mobilization disperses intraneural fluid in cervical nerve roots: A human cadaveric investigation. Musculoskelet. Sci. Pr..

[55] Martín Pérez S.E., Martín Pérez I.M., Sánchez-Romero E.A., Sosa Reina M.D., Muñoz Fernández A.C., Alonso Pérez J.L., Villafañe J.H. (2023). Percutaneous electrical nerve stimulation (PENS) for infrapatellar saphenous neuralgia management in a patient with myasthenia gravis (MG). Int. J. Environ. Res. Public Health.

[56] Ghasemi A., Zahediasl S. (2012). Normality tests for statistical analysis: A guide for non-statisticians. Int. J. Endocrinol. Metab..

